# Personality and High Blood Pressure Among Older Adults: State Anxiety as a Mediator

**DOI:** 10.1093/geroni/igaa057.1250

**Published:** 2020-12-16

**Authors:** Lisa Stone, Deborah Koh, Kendall Weber

**Affiliations:** 1 University of Colorado Colorado Springs, Colorado Springs, Colorado, United States; 2 University of Colorado at Colorado Springs, Colorado Springs, Colorado, United States

## Abstract

High blood pressure (BP) is a prevalent medical condition among older adults, and previous research has consistently found that the Five Factor Model (FFM) of personality relates to elevated BP. However, variables that affect this relationship have been unexamined. Given the strong association between personality and state anxiety in later life, this study examined state anxiety as a mediator between the FFM and elevated BP. Participants consisted of respondents in the 2018 wave of the Health and Retirement Study (N=5225) who completed FFM and state anxiety questionnaires and had their BP measured. First, correlations were computed between the FFM and systolic and diastolic BP. Relationships were insignificant between systolic BP and the FFM. Conscientiousness (r = -.04) and Neuroticism (r = .03) were significantly correlated with diastolic BP. Next, a series of mediation models were computed. Controlling for sex, the test of indirect effects found that the connection from Conscientiousness to diastolic BP was significantly mediated by anxiety, Sobel Z test = -2.44, p = .01. Additionally, controlling for sex, the connection from Neuroticism to diastolic BP was significantly and fully mediated by anxiety, Sobel Z test = 2.02, p = .04. Although small effect sizes, Conscientiousness and Neuroticism were related to diastolic BP but not systolic BP, indicating personality differentially affects types of BP. The mediating role of state anxiety suggests that it can be used as a point of intervention to lessen the negative impact of low Conscientiousness and high Neuroticism on high diastolic BP among older adults.

